# Research progress on adenosine in central nervous system diseases

**DOI:** 10.1111/cns.13190

**Published:** 2019-07-23

**Authors:** Ying‐Jiao Liu, Jiao Chen, Xun Li, Xin Zhou, Yao‐Mei Hu, Shi‐Feng Chu, Ye Peng, Nai‐Hong Chen

**Affiliations:** ^1^ College of Pharmacy Hunan University of Chinese Medicine Changsha China; ^2^ State Key Laboratory of Bioactive Substances and Functions of Natural Medicines Institute of Material Medical & Neuroscience Center Chinese Academy of Medical Sciences and Peking Union Medical College Beijing China; ^3^ Hunan Engineering Technology Center of Standardization and Function of Chinese Herbal Decoction Pieces Changsha China

**Keywords:** adenosine, Alzheimer's disease, cerebral ischemia, depression, epilepsy, Parkinson's disease, sleep disorders, structural modification

## Abstract

As an endogenous neuroprotectant agent, adenosine is extensively distributed and is particularly abundant in the central nervous system (CNS). Under physiological conditions, the concentration of adenosine is low intra‐ and extracellularly, but increases significantly in response to stress. The majority of adenosine functions are receptor‐mediated, and primarily include the A1, A2A, A2B, and A3 receptors (A1R, A2AR, A2BR, and A3R). Adenosine is currently widely used in the treatment of diseases of the CNS and the cardiovascular systems, and the mechanisms are related to the disease types, disease locations, and the adenosine receptors distribution in the CNS. For example, the main infarction sites of cerebral ischemia are cortex and striatum, which have high levels of A1 and A2A receptors. Cerebral ischemia is manifested with A1R decrease and A2AR increase, as well as reduction in the A1R‐mediated inhibitory processes and enhancement of the A2AR‐mediated excitatory process. Adenosine receptor dysfunction is also involved in the pathology of Alzheimer's disease (AD), depression, and epilepsy. Thus, the adenosine receptor balance theory is important for brain disease treatment. The concentration of adenosine can be increased by endogenous or exogenous pathways due to its short half‐life and high inactivation properties. Therefore, we will discuss the function of adenosine and its receptors, adenosine formation, and metabolism, and its role for the treatment of CNS diseases (such as cerebral ischemia, AD, depression, Parkinson's disease, epilepsy, and sleep disorders). This article will provide a scientific basis for the development of novel adenosine derivatives through adenosine structure modification, which will lead to experimental applications.

## 1 INTRODUCTION

Adenosine, short for adenine nucleoside, is chemically known as 6‐amino ‐9‐β‐d‐ribofuranosyladenine, and it is formed by the binding of adenine's N‐9 to D‐ribose's C‐1 via the β‐N9‐glucoside bond. Adenosine has strong effects in the coronary artery, and anti‐epilepsy, which is often used to treat cerebrovascular disorders, apoplexy sequelae, coronary insufficiency, angina, arteriosclerosis, and primary hypertension. Since energy utilization is ubiquitous,^1^ adenosine can be produced both intra‐ and extracellularly in tissues throughout the body, including the brain and heart. Adenosine is widely distributed in the central nervous system (CNS). Adenosine can be considered as a central excitatory and inhibitory neurotransmitter in the brain. Under ischemia, hypoxia, tissue damage, and other pathologic conditions, the degradation of ATP is increased. As a signaling nucleoside, adenosine plays a protective role by interacting with adenosine receptors when its extracellular concentration is increased.^2^

## 2 ADENOSINE STRUCTURE AND ITS MODIFICATION

**Figure 1:**
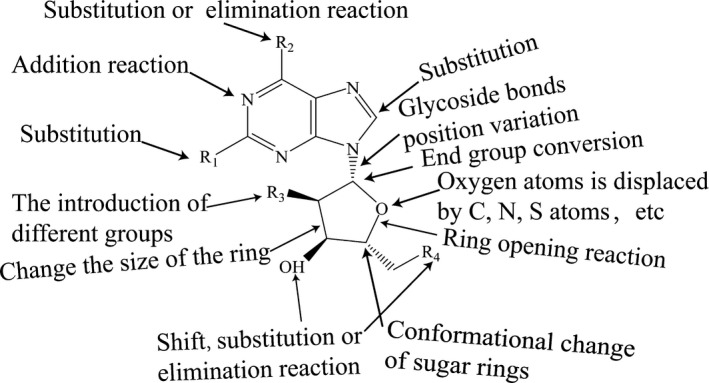
The modifying sites of adenosine. Adenosine derivatives are obtained by base modification, glycosyl modification, and simultaneous modification at multiple reaction sites of adenosine, which have different roles in central nervous system diseases

Adenosine contains multiple reactive sites of nucleoside molecules and thus has a strong “plasticity.” According to the classification of types of modification, modifications can be divided into base modification, glycosyl modification, and simultaneous basic‐glycosyl modification, such as the addition of one site, the substitution of two sites, the substitution or elimination reaction of six sites on the base, and the introduction of different groups on the 2′ site of the glycosyl (Figure 1). The main introduction sites are 2, 6, and 5′ on the bases and glycosyl in the treatment of central nervous diseases. The modification of two sites in bases is usually a substitution reaction to produce 2‐halogen products, such as 2‐chloro‐adenosine. The six site amino derivatives can be deaminated by deamination or hydrolyzed by HNO_2_ diazotization to form hydroxyl substitution derivatives, such as inosine. 2′ site derivatives in glycosyl with protective effects on nerve cells were obtained by conversion, substitution and elimination, such as adenosine cobalt amine. Other derivatives are listed in Table 1.

**Table 1:** Structural modification of adenosine and its derivatives

Description	Structural modification	Pharmacological action	References
Adenosine	R_1_=H,R_2_=NH_2_,R_3_=OH,R_4_=OH	/	/
N‐6‐cyclohexyl adenosine	R_1_=H,R_2_=NH_2_,R_3_=OH,R_4_= 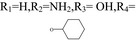	The damage of primary cultured cortical or hippocampal neurons was significantly reduced under the condition of oxygen‐glucose deprivation	3
2‐chloro‐adenosine	R_1_=Cl,R_2_=NH_2_,R_3_=OH,R_4_=OH	It induces astrocytes to apoptosis	4
Inosine	R_1_=H,R_2_=OH_2_,R_3_=OH,R_4_=OH	Inosine regulates depression‐like behavior, and binds to adenosine receptors to activate the intracellular ERK‐CREB signaling system	5
6‐(3‐phenylpropyl) amino‐2‐propyl sulfide adenosine	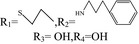	Antiplatelet aggregation activity	6
6‐(2‐furan methyl) oxy‐2‐propyl sulfide adenosine	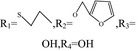	Antiplatelet aggregation activity	7
ATP	R_1_=H,R_2_=NH_2_,R_3_=OH,R_4_=O‐P～P～P	“energy currency,” as a signal molecule in the information delivering between nerve cells, it can improve the body's metabolism and energy source. ATP is a key factor that regulates neuronal support for axonal regeneration	8, 9
Cobamamide	R1=H,R2=NH_2_,R3=OH, R4=cobalamin	To reduce cerebral tissue damage and neuronal apoptosis induced by ischemia and anoxia injury	10

## 3 ADENOSINE PRODUCTION AND METABOLISM

**Figure 2:**
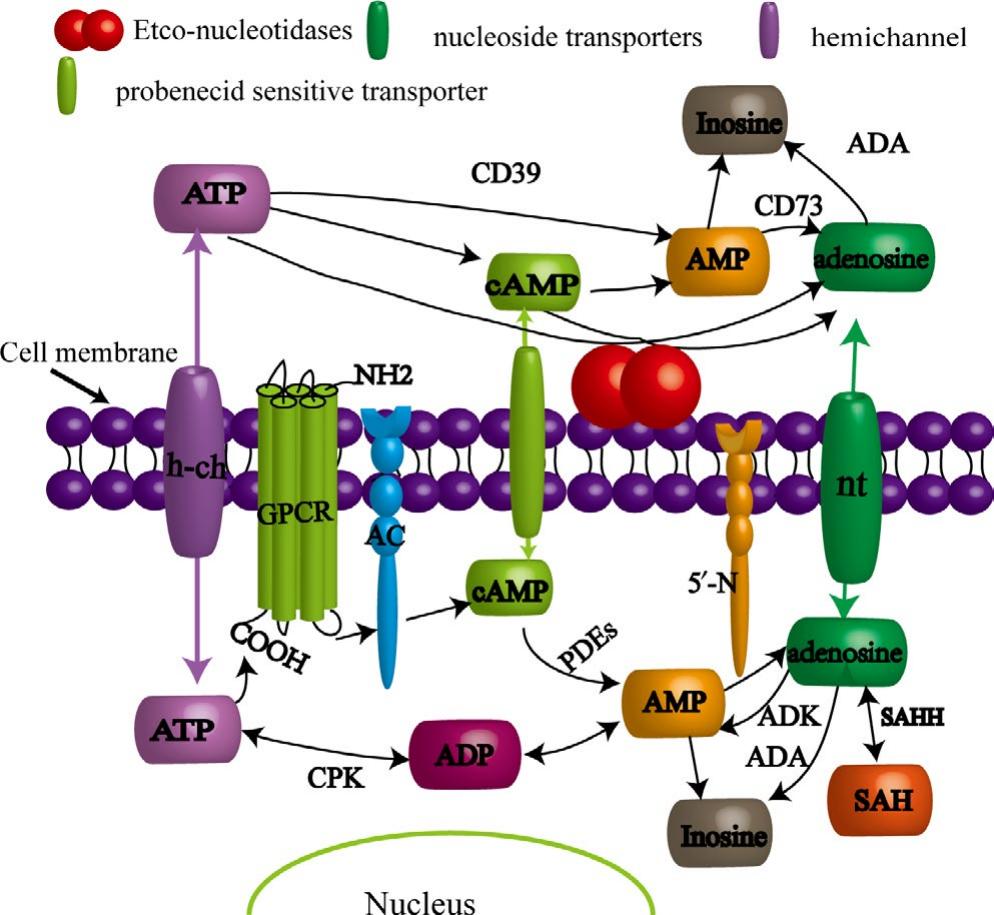
Production and metabolism of adenosine in and out of cells. In the physiological conditions, extracellular adenosine can not only be released by nerve endings or glial cells, but also from the metabolism of ATP. Adenosine cycle is completed by the mutual transformation of ATP and adenosine inside and outside the cell. In the stress state of ischemia, hypoxia, trauma and inflammation, neurons mainly increase the intracellular concentration of adenosine, while glial cells transport adenine nucleotides or adenosine out of the cell, thereby increasing extracellular the level of adenosine

Intracellular adenosine is produced mainly via three pathways: (a) When energy consumption increases, or the energy supply is relatively insufficient, ATP loses two phosphate groups and becomes AMP, which is converted into adenosine by removing phosphate groups under the action of the 5‐nucleotidase enzyme (5′‐N). (b) Adenine reacts with 1‐phosphate ribose to form phosphoric acid and adenosine.^11^ (c) S‐adenosyl‐l‐homocysteine (SAH) can be hydrolyzed into homocysteine and adenosine. Extracellular adenosine occurs mainly due to the transport of intracellular adenosine and the hydrolysis of extracellular adenine nucleoside. First, intracellular adenosine rapidly passes through the nucleoside transporters to maintain the extracellular adenosine concentration at a certain level. Second, ATP or ADP turns into AMP through CD39 or E‐NTPDase and then is hydrolyzed into adenosine by CD73 or 5′nucleotidase. In addition, the extracellular adenosine can be released by nerve endings or gliocytes (Figure 2). The physiological base level of extracellular adenosine is generally maintained at 0.04‐0.2 μmol/L.^12^

There are two main metabolic pathways of adenosine: (a) it becomes inosine under the action of adenosine deaminase (ADA) then generates hypoxanthine and hypoxanthine nucleotides through nucleosidases and finally becomes uric acid, which is an end product of purine derivatives in human metabolism.^13^ (b) Most adenosine enters the cell via a bidirectional balance transporter, and the adenosine kinase (ADK), which generates AMP by phosphorylation in central cells, only exists in the astrocytes. Adenosine kinase then forms ATP and completes the recycling of adenosine, which is also the main way to metabolise adenosine. Adenosine decomposition is carried out in the cell, and the extracellular adenosine must pass through the cell membrane into the cell through the above pathways of catabolism. Nitrobenzylthioinosine (NBTI) is an inhibitor of adenosine transport in cell membranes^14^ (Figure2).

Because of the rapid uptake and metabolism of adenosine, its balance is inefficient during times of stress. Adenosine does not exceed 1 μmol kg^−1^ in the brain under normal circumstances.^15^ However, adenosine degrades at a slower rate than those by which it is produced during ischemia, trauma, inflammation, and some other pathological conditions. This imbalance leads to a rapid increase in extracellular adenosine concentrations. However, adenosine has a short half‐life and this concentration change is temporary, and it is difficult to sustain as a protective mechanism against subsequent pathological conditions.

## 4 ADENOSINE RECEPTORS

Many physiological functions of adenosine are achieved through receptor mediation. The adenosine receptors belong to the G protein‐coupled receptor (GPCR) family. This family mainly includes four kinds of receptors, A1, A2A, A2B, and A3, of which A1R and A3R belong to the Gi family of G proteins while A2AR and A2BR are part of the Gs family.

A1R is a glycoprotein containing 326 amino acids with a relative molecular weight of 36 600. A1R has the highest affinity for adenosine, is coupled with pertussis sensitive G protein (Gi1~3,Go) and exists in all systems^16^. However, the expression of A1R is highest in the CNS, mainly distributed in the cerebral cortex, thalamus, hippocampus, basal forebrain, lateral hypothalamus, medulla oblongata, olfactory bulb and cerebellum, etc. The A1R is activated in the presynaptic membrane and can inhibit the activity of adenylyl cyclase (AC), reduce the content of cyclic adenosine‐3,5 monophosphate (cAMP), and regulate cAMP‐dependent protein kinase activity. In addition, A1R can also activate phospholipase C (PLC), and the latter can adjust the inositol phosphate metabolism of the cell membrane, increasing the content of inositol 1,4,5‐triphosphate (IP3) and diacylglycerol (DAG). Among these, IP3 can stimulate the release of Ca^2+^ from the intracellular calcium store and inhibit the N‐, Q‐, and P‐type calcium channels, which leads to the reduction in Ca^2+^ influx, inhibits the release of glutamate, and reduces the excitability of nerve conduction.^1^ In the postsynapse membrane, A1R becomes activated to open potassium channels and increase the outflow of K^+^, resulting in hyperpolarization of the membrane, thus reducing excitability and protecting neurons.^17^ In addition, A1R, when activated, can also open the ATP sensitive potassium channel (KATP) of neurons in the substantia nigra, increasing the outward current and decreasing the excitability of the membrane.^18^

The relative molecular weight of A2AR is 45 000, and it is mainly distributed in the brain areas rich in dopamine, such as the striatum globus pallidus, nucleus accumbens, olfactory tubercle, bulbus olfactorius, and nucleus nervi acustici. A2AR is also expressed in the cerebral cortex, amygdala, hypothalamus, hippocampus, thalamus, and cerebellum. When A2AR is activated, it is coupled with Gs protein in the peripheral tissues or Golf protein in the brain, and the protein kinase A (PKA) pathway is activated. Protein kinase A is also known as cyclic adenosine dependent protein kinase. Only the second messenger cAMP can activate PKA. Meanwhile, through the cAMP responsive element binding protein (CREB) known as cAMP‐PKA phosphorylated transcription factor, PKA interferes with nuclear factors‐activated‐κB (NF‐κB) and regulates gene expression.^19^ A2AR can also activate the mitogen‐activated protein kinases (MAPK), increase the production of collagen, and inhibit the peroxidation of neutrophils. Unlike A1R, adenosine promotes excitatory transmitter release through A2AR activation. In terms of blood vessels, A2AR also mediate vasodilatation and A2BR lead to a weak vasodilatation. Inhibition of platelet aggregation is mainly related to the A2A receptor.^20^

**Figure 3:**
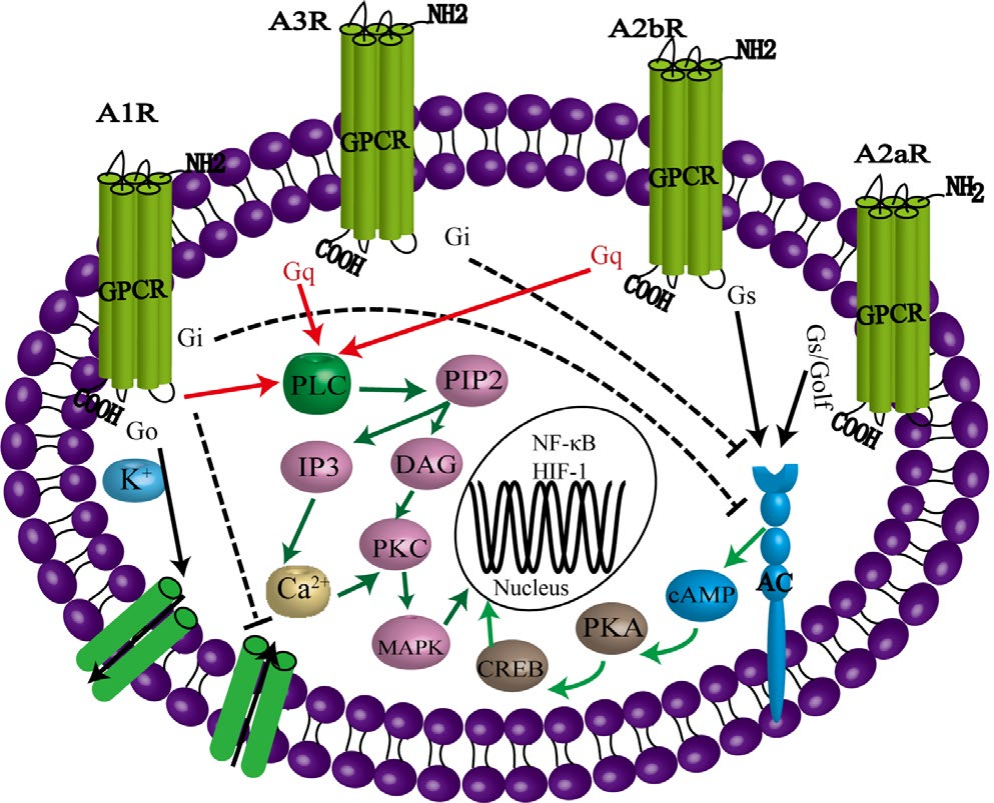
Signaling pathway of the AR. Adenosine receptors have different mechanisms of action after activation, thus playing a role in nerve protection or nerve injury

A2BR has a relative molecular weight of 36 350 and is mainly distributed in the neurons and gliocytes in the CA1 and CA3 regions of the hippocampus. There are also a few distributed in the thalamus, lateral ventricles, and striatum. When A2BR is activated, it can activate AC through Gs or PLC through Gq. Eckle et al^21^ believe that both pathways are activated simultaneously on human mast cells, resulting in IL‐4 secretion increasing significantly. Both A2AR and A2BR can be activated by adenosine, which reaches pathological concentrations higher than the physiological concentration after ischemia. A2BR can promote AC and cause increased intracellular cAMP, which promotes glycogenolysis and increases the energy supply of neurons. A3R is widely distributed throughout the brain but has the lowest density compared with other receptors, being mainly distributed in the hippocampus and cerebellum. Signaling pathways including Gi‐mediated AC inhibition and Gq‐mediated PLC activation are activated by A3R. A3R has different effects at different doses and administration times. Activation of A3R has both neuroprotective and neurotraumatic effects (Figure 3).

During the pathological circumstances of slight and short‐term ischemia, trauma, and inflammation, it is possible to improve the protein expression level of AR and increase the synthesis of receptor proteins; perhaps the membrane “spare receptor” is activated, or the transport of receptor proteins from the cytoplasm to the cell membrane is accelerated.^22^ The change in AR is sustained over a long duration of approximately 1‐3 or 7 days, during which adenosine is more likely to bind to its receptors that are synthesized by brain cells.

## 5 THE ROLE OF ADENOSINE AND ITS RECEPTORS IN CENTRAL NERVOUS SYSTEM DISEASES

### 5.1 Adenosine and cerebral ischemia

Cerebral ischemia is a common acute cerebrovascular disease caused by a transient lack of blood supply resulting in the onset of symptoms that are similar to the symptoms of cerebral hemorrhage or cerebral infarction. Patients with transient ischemic attacks may undergo cerebral infarction generally within 1‐5 years, and one‐third to two‐thirds of patients with cerebral infarction have had previous transient ischemic attacks. Cerebral ischemia is often characterized by the excessive increase in excitatory amino acids (EAAs), reactive astrogliosis, and nerve damage. As an endogenous neuroprotective agent, adenosine exhibits homeostasis and neuroregulation effects on the changes during ischemia, hypoxia, and stroke.^23^

#### 5.1.1 Excitatory amino acid (EAA)

**Figure 4:**
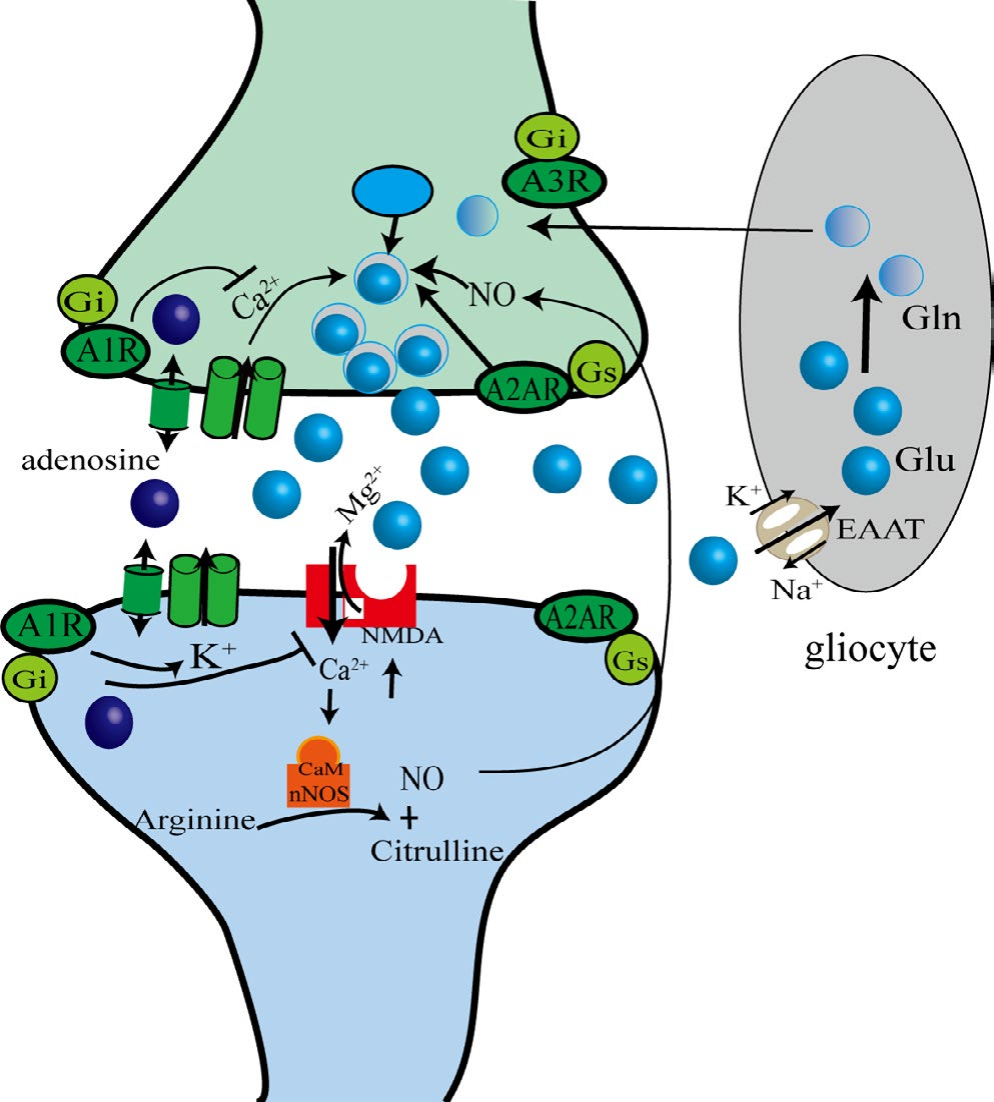
Related mechanism of adenosine and release of excitatory amino acids

The toxicity of excessive EAAs, such as glutamic acid and aspartate, is an important cause of neuronal death in cerebral ischemia. By activating A1R, the release of EAA can be inhibited to protect nerve cells, and the mechanism may be as follows: In presynaptic membranes, A1R reduces calcium influx by inhibiting N or Q‐type Ca^2+^ channels and reduces phospholipase activity, while decreased calcium influx can inhibit EAA release. In the postsynaptic membrane, A1R can open potassium channels, which can lead to the hyperpolarization of the membrane by increasing the K^+^ outflow, inhibiting the excitations of neurons, and protecting the neurons. A1R and glutamate, n‐methyl‐d‐aspartic acid (NMDA) receptors have a similar distribution in the brain. Glutamate is mainly stored in the synaptic vesicles after uptake by VGLUTs on the vesicles and is affected by the concentration of Ca^2+^ and nitric oxide (NO). Nitric oxide is generated by arginine catalyzed by neuronal nitric oxide synthase (nNOS), in which Ca^2+^/CaM is a necessary condition for nNOS activation. Hedison et al^24^ think that the immune response to CaM and nNOS can determine the degree and duration of brain injury. They can also act as a signaling molecule into the cells' interior and can quickly penetrate the cell membrane and act on neighboring cells. Once released, glutamate functions in the synapse through the mediation of ionic or metabolic receptors. For example, AMPA receptors lead to depolarization of the postsynaptic membrane, causing the blocking channel Mg^2+^ to be removed, and glutamate binds to the NMDA receptor, causing the Ca^2+^ channel to be opened. The function of glutamate is quickly terminated by the excitatory amino acid transporters (EAATs) located in the cytoplasmic membranes of glia and neurons 25 (Figure4).

A2AR can directly mediate EAA release, and in addition, it can indirectly promote EAA release by interacting with other receptors, such as dopamine receptors, A1R, and glutamate receptors. Moreover, the protective effect of selective A2AR antagonists on cerebral ischemia is related to the dose. Low doses of selective A2AR antagonists have a neuroprotective effect, but the protective effect disappears at an increased dose. The possible reasons are that a high dose of selective A2AR antagonists may block other adenosine receptors, such as A1R, and other possibilities are its peripheral effects on blood pressure reduction, heart rate disruption, and platelet aggregation, which lead to nutrition deficiency in brain injury sites. But its priority to block the A2AR on postsynaptic membranes can inhibit NMDA toxicity thus protect the nerve^26^. Popoli et al^27^ think that the selective A2AR antagonist CSC has a neuroprotective effect during the first 15 minutes after cerebral ischemia in rats, but this effect will disappear after 2 weeks of continuous treatment with the same dose of CSC. Thus, the medication duration is another factor influencing the neuroprotective effect of the selective A2AR antagonist.

Therefore, adenosine can inhibit the release of glutamate and aspartate after ischemia and reduce the cytotoxic effects of EAA, thereby protecting neurons.

#### 5.1.2 Reactive astrogliosis

Reactive astrogliosis is a repair response that is characterized with astrocytes hypertrophy, protuberant elongation, glial fibrillary acidic protein (GFAP) expression and astrocyte proliferation after brain damage. The activation of different adenosine receptors has different effects on reactive gliosis. A1R and A3R have inhibitory effects on reactive gliosis, while A2R promotes glia reactions. A1R can accelerate the apoptosis of RCR‐1 astrocytoma in rats,^28^ and A3R can also inhibit reactive gliosis by inducing apoptosis of astrocytes. Since the adenosine analogue 2‐chloro‐adenosine (2‐CA) is not sensitive to A1R and A2R antagonists, this may be mediated by A3R through cysteine kinase‐3 pathways, or it may be related to the decrease in the concentration ratio between SAH and S‐adenosyl methionine (SAM), which triggers apoptosis.^29^ A2R may indirectly promote reactive gliosis by interacting with other cytokines, such as tumor necrosis factor‐alpha (TNF‐α), basic fibroblast growth factor (bFGF).

#### 5.1.3 Trophic action of nerves

Astrocytes can secrete a large number of neurotrophic factors and cytokines with neuroprotective effects, including nerve growth factor (NGF), S‐100B, neurotrophic factor 3 and 4 (NT3 and NT4), brain‐derived neurotrophic factor (BDNF) and vascular endothelial growth factor (VEGF), IL‐6 and selective chemokines (CCL2). Nerve growth factor plays a neuroprotective role in cerebral ischemia, it is secreted mainly by astrocytes next is microglia, and is related to A1R and A2aR, respectively. As one of the factors in the S‐100 family, S‐100B is a Ca^2+^ binding protein mainly secreted by astrocytes and plays important roles in cell proliferation, differentiation, and protein phosphorylation, which is mediated by adenosine A1R. After binding to its receptor, S‐100B can induce NF‐κB nuclear translocation, stimulate the expression of Bcl 2, and promote the recovery of neurons and the growth of axons.

### 5.2 Adenosine and Alzheimer's disease

Alzheimer's disease (AD) was first discovered and reported in 1906 by the German physician Alois Alzheimer. It is a common chronic progressive central neurodegenerative disease in the elderly and characterized with neuritic plaques (NP), neurofibrillary tangles (NFT), and neuronal death. There are many pathogenesis theories including cholinergic theory, free radical theory, β‐amyloid theory, inflammation and immune theory, oxidative stress theory, and Tau protein gene theory; Yan Rong et al^30^ proposed an adenosine receptor balance theory and believed that maintaining the balance of the adenosine receptor might be a new and important way to prevent and treat AD.

During the pathogenic process of AD, the balance of adenosine receptors is disrupted, and mainly manifested as A1R expression decrease and A2AR expression increase, as well as disruption in inhibition and excitation processes, which eventually leads to cognitive dysfunction.^31^ Therefore, the balance of adenosine receptors can play a neuroprotective role by activating A1 receptors,^32^ inhibiting A2a receptors,^33^, ^34^ restoring the function of the cholinergic system, and improving the hippocampal synaptic plasticity and neurotrophic factors.^35^ Alzheimer's disease starts with the lack of synaptic function, and adenosine A2AR is mainly located in the synapses that control synaptic plasticity. So A2AR antagonists can repair the early synaptic and memory dysfunction of AD.^36^ Pagnussat et al^37^ believe that A2ARs are necessary and sufficient for triggering memory impairment, and A1R may selectively participate in controlling memory impairment driven by cholinergic processes. Nabbi‐Schroeter et al^38^ suggested that caffeine, a nonselective antagonist of adenosine receptors, may protect against neuronal degeneration and death caused by β‐amyloid by blocking A2AR and observed that the A2BR antagonist has a certain protective effect against AD. In addition, there is an association between isoflurane exposure and spatial memory impairment, and declines in NR2B levels and increases in Aβ and P‐tau levels may be achieved by activating A1AR.^39^ However, Yao et al^40^ believed that Aβ_(1‐42) induced AD through the CD73 specific inhibitor APCP (α,β‐methylene adenosine‐5′‐diphosphate) and that it can significantly enhance the learning ability and working‐memory capacity and increase the central excitability of AD mice, which may be related to the reduction of the content of adenosine outside the hippocampal cells.

In adulthood, new neurons are created in the dentate gyrus of the hippocampus and subventricular zone, two areas of the brain associated with AD. Disruption of this process can lead to neurodegenerative diseases, including AD. Based on a genetic correlation analysis, adenosine A2AR was significantly correlated with hippocampal volume, adenosine rs9608282 small alleles being associated with a greater hippocampal volume and improved memory.^41^ In terms of other receptors, caffeine has a protective effect on the amyloid process by inhibiting the A3R‐mediated internalization of the β‐amyloid precursor.^42^ Thus, the adenosine receptor balance theory has been increasingly accepted in AD.

### 5.3 Adenosine and depression

Depressed mood is the main clinical symptom of depression disorder. The hypothalamus, as the center of the neuroendocrine system, integrates neuromodulation and humoral regulation, and regulates the human body temperature, food intake, and endocrine activities extensively. Through close contact with the limbic system, the hypothalamus participates in emotional responses. Many studies have reported on depression and adenosine. In 2001, Berk showed that the function of the adenosine A2a receptor was weakened in platelets of patients with depression. In 1999, Elgun found a decline of adenosine deaminase (ADA) activity in the plasma of patients with major depression, and it had a negative correlation with the severity of depression, which indirectly suggested that adenosine may be involved in the pathological changes of major depression. Adenosine receptors also play a role in the balance theory during depression. Lindquist et al^43^ think that adenosine A1R and A2AR are involved in the antidepressant effect of adenosine. Adenosine A2AR is upregulated in the cortex by early febrile seizures, thus inducing depressive behavior in adult rats.^44^ It was demonstrated that caffeine, a nonselective A2AR antagonist, can prevent chronic depression caused by excision of the bilateral olfactory bulb.^45^ In addition, the adenosine level is negatively correlated with affective disorder and purine is not adequately balanced in these patients.^46^ Adenosine and inosine regulate depression‐like behavior in adult rodent models, and inosine is the adenosine metabolism product that binds to adenosine receptors and activates the intracellular ERK‐CREB signaling pathway.^5^

The equilibrium effect of A1R and A2AR regulates the release of monoamine neurotransmitters in the antidepressant effect of sleep deprivation, especially 5‐HT. The mechanism is that adenosine binds to A1R to inhibit the release of 5‐HT and at the same time integrates with A2AR to promote the release of 5‐HT. The concentration of adenosine is reduced significantly after chronic stress, thus breaking the balance of its binding to A1R and A2AR. At low concentrations, adenosine mainly activates A1R, thereby inhibiting the release of 5‐HT, leading to a decrease in 5‐HT concentration in the hypothalamus and the occurrence of depression.^47^ Therefore, adenosine may not be directly involved in the antidepressant effect of sleep deprivation but affects its pathogenesis by regulating 5‐HT release.

### 5.4 Adenosine and Parkinson's disease

Parkinson's disease (PD) is a common neurodegenerative disease. The main pathological change is the degeneration and death of dopaminergic (DA) neurons in the substantia nigra of the midbrain, which leads to a significant decrease in the content of DA in the striatum and the occurrence of acidophilic inclusion bodies (Lewy bodies) in the cytoplasm of surviving neurons in the substantia nigra.^48^

The basal ganglia have two output pathways, direct and indirect, that control smooth and coordinated movement, and they converge in the pallidum and the substantia nigra. The efferent neurons of the indirect pathway are GABA/SP/DYN, which are distributed with excitatory D1‐type DA receptors. The efferent neurons of the indirect pathway are GABA/ENK, with inhibitory D2‐type DA receptors. Indirect pathways exert inhibitory effects on neurons in the thalamocortical system, rather the opposite of direct pathways. Adenosine promotes the indirect pathway by regulating A2AR in the striatum and globus pallidus. Parkinson's disease is caused by the degeneration of dopaminergic neurons in the pars compacta of the substantia nigra, which weakens their excitatory effect on the striatum GABA/SP/DYN neurons. These events cause the direct pathway to be relatively inhibited, while the inhibitory effect of DA is lost by GABA/ENK. This phenomenon results in the relative activation of indirect pathways, enabling both pathways to enhance the activity of the complex of internal segments of the globuspallidus (Gpi) and substantia nigra pars reticulate (SNr), finally resulting in suppression of the thalamus, decreased activity in the cortex motor area, and eventually resulting in a series of symptoms of PD. The pathogenesis of PD and adenosine A2AR are correlated. Since adenosine A2AR is mainly distributed postsynaptically, only the small number distributed presynaptically mainly inhibit calcium influx, thereby inhibiting the release of neurotransmitters and playing a negative feedback regulation role. It is believed that A2AR has a unique control of the maintenance and extraction of working memory in the globus pallidus and medial prefrontal cortex neurons, and A2AR antagonists can alleviate the cognitive impairment of PD.^49^ In addition, A2AR antagonists can also reduce l‐dopa induced dyskinesia.^50^ Therefore, adenosine A2AR antagonists play an important role in the treatment of PD.

Adenosine A2AR also interacts with other neurotransmitters in PD. For example, adenosine A2AR and D2 receptors may have mutual antagonistic effects, and adenosine A2ARs are partially dependent on D2 receptors to play a role. 5‐HT 1A/1B receptor agonists and adenosine A2AR antagonists were combined to improve motor dysfunction by inhibiting excessive DA release in serotonin neurons and increasing its influence on the indirect pathway of the striatum efferent nerve.^51^ Adenosine A2AR interacts with the glutamate metabotropic Glu5 receptor (mGlu5 receptor) in glutamate release, and co‐localization exists. The effects of glutamate release by the mGLu5 selective agonist can be blocked by A2AR antagonists in the striatum. Both A2AR antagonists and mGLu5 antagonists, when used alone or in combination, can significantly reduce the dyskinesia caused by 6‐hydroxydopamine (6‐OHDA) damage,^52^ and the combined use of the two could also increase the inhibition of D2 signals of GABA neurons in the globus pallidus.^53^ Adenosine A2AR has synergistic effects with the M receptor, showing the effect of superposition.^54^ That is, A2AR can stimulate the release of acetylcholine in striatal neurons, and the cholinergic nerve fiber endings can also regulate the release of acetylcholine by negative feedback from muscarinic (M) receptors in the presynaptic membrane. The M receptor antagonist can resist the action of A2AR agonist.^55^ Therefore, combined medication may be an effective measure to treat PD.

### 5.5 Adenosine and epilepsy

Epilepsy is a chronic and recurrent brain dysfunction syndrome that is caused by the abnormal discharge of neurons in the brain. Epilepsy is clinically manifested as a whole body tonic clonus attack, and during the seizure, the EEG shows typical explosive multi‐spinous waves, and spine‐slow waves. The main pathogenesis is glial cell proliferation, adenosine dysfunction, abnormal nerve conduction pathways, selective neuron cell loss, nerve inflammation, and mossy fiber bud phenomenon.

#### 5.5.1 Astrocyte

Astrocytes are large glial cells that are the most widely distributed glial cells in the brain of mammals. Astrocytes also make up the largest volume of glial cells with a wide range of intercellular gap connections. Astrocytes have a variety of neurotransmitter receptors, such as excitatory glutamate receptor, inhibitory γ‐aminobutyric acid receptor (GABAR), acetylcholine receptor, dopamine receptor, and so on. Neurotransmitters released by neurons can also act on glial cells and cause complex physiological responses. Astrocytes play an important role in the formation, development, and treatment of epilepsy. Astrocytes are the main source of adenosine in synapses and play a regulatory role.^56^ Pascual et al^57^ found that there was an inhibition function due to the lack of adenosine during synaptic transmission by knocking out the gene in mouse astrocytes, and it proved for the first time that astrocytes can release ATP, thereby regulating the content of adenosine in synapses. Li et al^58^ believe that astrocytosis and adenosine homeostasis dysfunction are pathological features of the epileptic brain, and this contributes to the occurrence of epileptic seizures.

#### 5.5.2 Adenosine steady‐state

Adenosine kinase converts adenosine into AMP in astrocytes, and the intracellular adenosine concentration then decreases. Extracellular adenosine enters the cell through nucleoside transporters to maintain the balance of adenosine concentration inside and outside the cell. High concentrations of ADK cause low concentrations of adenosine; conversely, low concentrations of ADK correspond to high concentrations of adenosine. Changes in ADK can cause significant changes in adenosine levels but have little effect on energy metabolism. Therefore, ADK is a key factor in astrocytes that determines the concentration of adenosine in synapses and regulates synaptic excitatory transmission. The increased activity of ADK reduces the content of adenosine in synapses and promotes the development of epilepsy. Inhibiting ADK is expected to reveal new measures to prevent epilepsy.

#### 5.5.3 Glutamic acid

Glutamate has the highest content of amino acids in the CNS and is one of the explicit EAAs. Glutamate has a wide and strong excitatory effect on the cerebral cortex and is closely related to the triggering and spreading of epileptic discharges. Ca^2+^ is an important second messenger, and calcium signaling in astrocytes is mainly controlled by receptors. The release of glutamate is related to adenosine binding to its receptor. When adenosine binds to A1R, it inhibits the release of excitatory transmitter glutamate by inhibiting the N‐type voltage‐gated calcium ion channel. When adenosine binds to A2AR, it increases intracellular calcium ion concentration through the protein kinase A pathway, thereby stimulating the release of glutamate.^59^ Therefore, activation of adenosine A1R or inhibition of adenosine A2AR can inhibit the release of glutamate.

#### 5.5.4 Epigenetic modification

Epigenetic modifications include changes in DNA methylation that lead to the change in gene expression. Therefore, there may be a basis of epileptogenesis by inducing permanent changes in neural excitability.^60^ The main mechanism is that the methyl group is provided by S‐adenosyl‐l‐methionine(SAMe) and selectively added by cytosine of C and G nucleotides of DNA under the catalysis of DNA methyltransferase(DNMTs), and s‐adenosine homocysteine (SAH) produces adenosine and homocysteine by hydrolytic enzyme.^61^ Therefore, adenosine can inhibit or reverse these changes, such as adenosine not being cleared in time from the synaptic cleft, and the treatment of exogenous adenosine can inhibit the process of DNA methylation and thus inhibit the occurrence of epilepsy. The epigenetic function of endogenous adenosine was determined by inducing DNA hypomethylation due to biochemical interference into the methylation pathway.^62^ Exogenous adenosine can reverse the DNA methylation process in temporal lobe epilepsy of rats, inhibit the mossy fiber sprouting in the hippocampus, and control the occurrence of epilepsy.

#### 5.5.5 Gamma‐aminobutyric acid receptor

**Figure 5:**
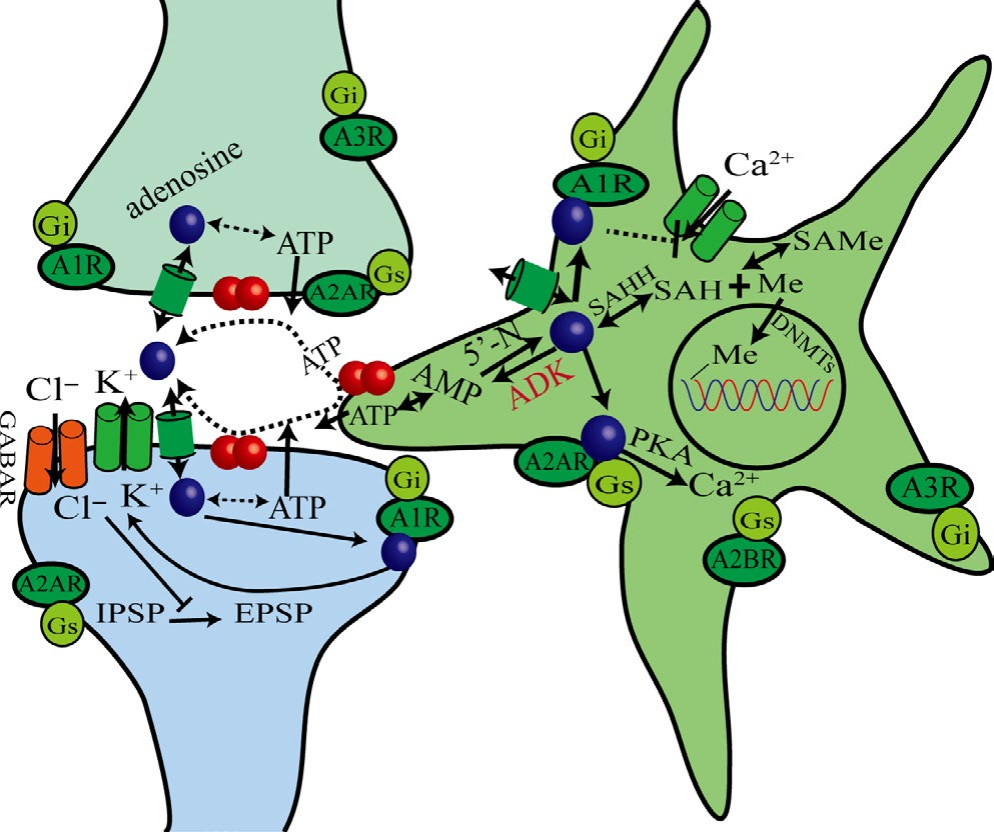
The mechanism of action of adenosine in epilepsy. Adenosine therapy for epilepsy was summarized from the aspects of astrocytes, ADK induced the changes of adenosine concentration, changes in DNA methylation, GABAAR

GABA is an important inhibitory neurotransmitter in the CNS. Its main function is inhibitory postsynaptic potential, which can make the postsynaptic membrane in its hyperpolarization state and reduce the activity of neurons by combination with its receptor. Cl^−^ flux mediated by GABAR in postsynaptic neurons of adult brain causes the hyperpolarization of neurons and inhibition of nerve conduction. When the concentration of Cl^−^ is reversed in neurons during epileptic seizures, the signal of GABA‐ergic changes from hyperpolarization (IPSP) or inhibitory to depolarization (EPSP) or excitatory. Hyperpolarization and depolarization are mainly caused by the opening of different ion channels on the cell membrane, which leads to the movement of differently charged ions across the membrane, so that the positive and negative charges in the cell membrane are changed, resulting in a change of membrane potential; this change in potential determines the functional state of the cell, that is excitation or inhibition. Drugs that block adenosine receptors can prolong the duration of epileptic seizures. This mechanism may be related to the activation of adenosine receptors and potassium ion channels by endogenous adenosine,^63^ thereby increasing membrane conductance and inhibiting the transition from hyperpolarization to depolarization (Figure 5).

Therefore, adenosine inhibits the pathogenesis of epilepsy through astrocytes, ADK, glutamate, epigenetic gene modification, the gamma‐aminobutyric acid receptor and other pathways, which will provide new ideas and measures for the treatment of epilepsy.^64^

### 5.6 Adenosine and sleep disorders

The “sleep substance,” adenosine, is an effective endogenous sleep‐promoting factor which accumulates in the brain during wakefulness and induces physiological sleep.^65^ Among the four adenosine receptors, the role of A2AR is predominant in sleep regulation, whereas A1R contributes to sleep induction in a region‐dependent manner but may not be absolutely necessary for sleep homeostasis.^66^ In normal physiological conditions, endogenous adenosine promotes sleep regulation of neurons by acting on A1R and A2AR to stimulate sleep or inhibit arousal. The main acting sites are basal forebrain, subarachnoid space, hypothalamic nucleus and brainstem. Cholinergic neurons in the basal forebrain, histaminergic neurons, and hypocretin in the hypothalamus, monoaminergic neuron (dopamine, 5‐hydroxytryptamine, norepinephrine) in the brainstem send out fibers that constitute the ascending reticular activation system (ARAS), which plays a role in cortical arousal.^67^ In addition, adenosine can also activate GABA neurons by reducing GABA inputs in the ventrolateral preoptic nucleus (VLPO) for sleep induction, and inhibit glutamate neurons in cerebral cortex, striatum, hippocampus, and other brain regions to achieve the coordination between different sleep behaviors.^68^

## 6 ADENOSINE TREATMENT

Manipulation of endogenous adenosine level is used in the treatment of CNS disorders. For example, adenosine A1R agonists or ADK inhibition can effectively prevent or reduce the onset of CNS diseases. However, exogenous adenosine therapy is not the main treatment method,^69^ due to the side effects of adenosine in the cardiovascular system and the hepatotoxicity of ADK inhibitors, as well as the fact that exogenous adenosine is quickly absorbed and metabolized after entering the human body by the high affinity nucleoside transporters located on red blood cells, capillary endothelial cells, and smooth muscle cells.^70^ However, the ultimate goal of these approaches is to increase local adenosine levels in the brain while avoiding side effects and damage to other organs. Therefore, adenosine metabolic balance has become the developmental direction of adenosine synergistic therapy in brain.

### 6.1 Silk that controls adenosine release

The continuous release of adenosine can be controlled by planting silk membranes wrapped with adenosine around the hippocampus, which can effectively control the onset of epilepsy.^71^

### 6.2 ADK expression control

Adenosine can be released from human mesenchymal stem cells and mouse embryonic stem cells with endogenous ADK gene knocked out after being implanted into the hippocampus.^72^ Theofilas used an adenovirus eight gene expression system to knock out the ADK gene, the ADK level of glial cells in the hippocampus decreased, and the occurrence of epilepsy was controlled.^73^

### 6.3 Adenosine receptors

When the adenosine A1R gene was knocked out in mice, a ketogenic diet could not exert effects on epilepsy, indicating that a ketogenic diet inhibited the generation of epilepsy through the excitatory effect of adenosine A1R.^74^ Yang et al^75^ also demonstrated that a ketogenic diet significantly increased the tolerance to cerebral ischemia in mice, which may be achieved by increasing the level of extracellular adenosine in the ischemic area and may be mediated by A1 receptors. Researchers also found that the c‐fos immune response activity in KO mice was higher than in WT mice by knocking out adenosine A2A receptors in the anterior cingulate and amygdala, thus causing defects in social active behavior (social withdrawal), such as in autism and depression.^76^

### 6.4 Trophic action of nerve

As a metabolite of adenosine, inosine can promote axonal regeneration, mainly by activating intracellular serine/threonine kinases, thus upregulating the key factor GAP‐43 protein required for axonal regrowth and acting as a neural growth factor.^77^

### 6.5 Improve blood rheology

After the knockout of the A2AR gene in mice, platelet aggregation was enhanced. Therefore, the activation of A2AR could improve haemorheology, inhibit platelet aggregation, and promote thrombolysis.^78^

### 6.6 Excitatory cytotoxicity and Ca^2+^ alleviation

Low and medium concentrations of adenosine can activate adenosine A1R and inhibit the release of glutamate. Medium and high concentrations of adenosine can activate adenosine A2AR and promote the release of glutamate. Adenosine A2AR has an inhibitory effect on A1R at medium and high concentrations, and the inhibitory effect increases with concentration.^79^

## 7 SIDE EFFECTS OF ADENOSINE

Adenosine has a few side effects. The most common side effects are flushing, discomfort in the throat, neck, jaw, upper limbs and gastrointestinal tract, breathing difficulties, and dizziness. Because adenosine has a very short half‐life, these side effects usually do not require intervention and disappear quickly, which are easily accepted by patients. Adenosine is applicable to patients who cannot exercise for various reasons or cannot achieve the target amount of exercise.

## 8 DISCUSSION

In conclusion, with the growing use of adenosine in the CNS, an increasing number of derivates have been invented with adenosine structure modified. Modifying the adenosine structure according to its pharmacological action provides a new direction for the design and development of new drugs. At the same time, the adenosine mechanisms are related to disease types, lesion location, and receptor distribution. For example, the cortex and striatum are the main infarction sites of cerebral ischemia, and the adenosine receptors in this area are mainly A1R and A2AR. Cerebral ischemia mainly manifests as decreased expression of A1R and increased expression of A2AR, and the effect of inhibition mediated by A1R is weakened and the effect of excitation mediated by A2AR is enhanced.^80^ Similar mechanisms exist in AD, depression, and epilepsy.^81^ Therefore, adenosine receptor homeostasis in the brain is the key to the treatments of these diseases. However, the main cause of PD is the significant decrease in DA content in striatum, which is enriched with adenosine A2AR; thus, adenosine A2AR antagonists play an important role in the treatment of PD.^82^ The pathogenesis of CNS diseases is directly or indirectly related to adenosine receptors distributed at the site of the disease. The concentration of adenosine rises sharply in the extracellular fluid of the synaptic space, reaching over 200 times or even 1000 times as high as the normal level under stress. High concentrations of adenosine may maintain the homeostasis of nerve cells, delay or reduce cell death, and play a neuroprotective role through the abovementioned multiple functions. However, the concentration of adenosine is only temporarily elevated and is quickly metabolized. At the same time, a high concentration of adenosine activates A2AR, and the concentration of EAA also rises sharply, initiating a series of pathophysiological processes that eventually lead to the necrosis or apoptosis of nerve cells.^83^ As low concentrations of adenosine can activate A1R and inhibit the release of EAAs, A2AR is conversely activated with an increase in adenosine concentration, and A2AR can block heteromeric A1R through a receptor‐receptor allosteric trans‐inhibition.^79^ Therefore, adenosine A2AR antagonists are very important for the protection of the CNS.

In the pathological conditions of ischemia, hypoxia, trauma, and inflammation, the extracellular adenosine concentration increases rapidly, resulting in an imbalance of intracellular and extracellular concentrations of adenosine. Adenosine has a short half‐life, adenosine concentration is difficult to sustain the protective effect during pathological conditions. There are many ways to manipulate the endogenous adenosine concentration including adenosine agonists administration, ADK, ADA and extracellular enzymes (such as CD39 and CD73) regulation and the use of dipyridamole to inhibit the adenosine transporter.^84^ Exogenous adenosine can also be used to increase the adenosine concentration,^85^ Yuan et al^86^ found that the effect of preconditioning with adenosine on brain ischemic tolerance in rats. Perrier et al^87^ think that the main effects of adenosine were to decrease neurotransmitter release probability and to attenuate short‐term depression mechanisms. But this use is relatively rare, and it needs to be further examined. At the same time, whether adenosine receptor balance or intracellular and extracellular adenosine concentration balance has a key role in the treatment of CNS diseases remains unclear. The specific mechanisms remain to be further studied.

## CONFLICT OF INTEREST

All authors declare no conflict of interest.
